# Diesel Exhaust Particle Exposure *In Vitro* Alters Monocyte Differentiation and Function

**DOI:** 10.1371/journal.pone.0051107

**Published:** 2012-12-07

**Authors:** Nazia Chaudhuri, Hannah Jary, Simon Lea, Naimat Khan, Katie C. Piddock, David H. Dockrell, Ken Donaldson, Rodger Duffin, Dave Singh, Lisa C. Parker, Ian Sabroe

**Affiliations:** 1 Academic Unit of Respiratory Medicine, School of Medicine and Biomedical Sciences, University of Sheffield, Sheffield, United Kingdom; 2 Education and Research Centre, University Hospital of South Manchester NHS Trust, Manchester, United Kingdom; 3 The University of Edinburgh Centre for Inflammation Research, Queens Medical Institute, Edinburgh, United Kingdom; Leiden University Medical Center, The Netherlands

## Abstract

Air pollution by diesel exhaust particles is associated with elevated mortality and increased hospital admissions in individuals with respiratory diseases such as asthma and chronic obstructive pulmonary disease. During active inflammation monocytes are recruited to the airways and can replace resident alveolar macrophages. We therefore investigated whether chronic fourteen day exposure to low concentrations of diesel exhaust particles can alter the phenotype and function of monocytes from healthy individuals and those with chronic obstructive pulmonary disease. Monocytes were purified from the blood of healthy individuals and people with a diagnosis of chronic obstructive pulmonary disease. Monocyte-derived macrophages were generated in the presence or absence of diesel exhaust particles and their phenotypes studied through investigation of their lifespan, cytokine generation in response to Toll like receptor agonists and heat killed bacteria, and expression of surface markers. Chronic fourteen day exposure of monocyte-derived macrophages to concentrations of diesel exhaust particles >10 µg/ml caused mitochondrial and lysosomal dysfunction, and a gradual loss of cells over time both in healthy and chronic obstructive pulmonary disease individuals. Chronic exposure to lower concentrations of diesel exhaust particles impaired CXCL8 cytokine responses to lipopolysaccharide and heat killed *E. coli*, and this phenotype was associated with a reduction in CD14 and CD11b expression. Chronic diesel exhaust particle exposure may therefore alter both numbers and function of lung macrophages differentiating from locally recruited monocytes in the lungs of healthy people and patients with chronic obstructive pulmonary disease.

## Introduction

Inhalation of small particulate matter is a major cause of airway inflammation, and rising air pollution levels are associated with elevated mortality and increased hospital admissions in individuals with respiratory diseases such as asthma and chronic obstructive pulmonary disease (COPD) [1], [2], [3]. Diesel Exhaust Particles (DEP) are a major particulate matter of air pollution. Acute and chronic inhalation of DEP in healthy individuals, and those with pre-existing respiratory disease, results in respiratory toxicity with consequent development of lung oedema, infiltration of polymorphonuclear leukocytes, and the production of proinflammatory cytokines and reactive oxygen species (ROS) [4], [5], [6], [7]. Consequently these can lead to deterioration of pre-existing respiratory disease [8] and development of symptoms [9].

The adverse effects of DEP on alveolar macrophages (AMs) have been extensively studied [10], [11]. AMs represent one of the first lines of defence against environmental and pathogenic stimuli, and have lower functional responsiveness to environmental particles compared to monocytes [12]. AMs are heavily implicated in the pathogenesis of COPD [13] and are key players in orchestrating the inflammatory response. The active inflammation that occurs in asthma and COPD leads to the recruitment of circulating monocytes to the alveolar wall, with the subsequent replacement of resident AMs [14]. In addition, exposure to particulate matter has been shown to cause the release of monocytes from the bone marrow [15], [16]. We have demonstrated that monocytes avidly phagocytose DEP ( Figures S1 and S2), however, there are no studies to date investigating the chronic effects of DEP on monocytes from individuals with COPD and their subsequent fate and activation.

The existence of chronic inflammation as is observed in individuals with COPD may alter the functional responses of these cells to varied stimuli. Indeed, alveolar macrophages from COPD individuals show reduced phagocytosis of apoptotic epithelial cells compared to non smokers [17] and monocyte derived macrophages (MDMs) from people with COPD also show reduced phagocytosis in response to pathogens [18]. These findings could have major implications for airway responses to inhaled pathogens and the subsequent development of chronic inflammation [19].

**Table 1:** Patient Demographics.

	Healthy n = 7	COPD n = 8
Sex (Male/Female)	4/3	5/3
Age (years)	66 (44–69)	62 (51–68)
FEV_1_ (L)	3.14 (2.45–4.36)	1.77 (1.38–2.97)
FEV_1_ predicted (%)	101 (91–126)	68 (57–81)
FEV_1_/FVC %	78 (70–81)	63 (34–84)
Pack year history	0	26 (13–60)
Inhaled corticosteroid use (%)	0	50
Current smokers (%)	0	50

**Figure 1:**
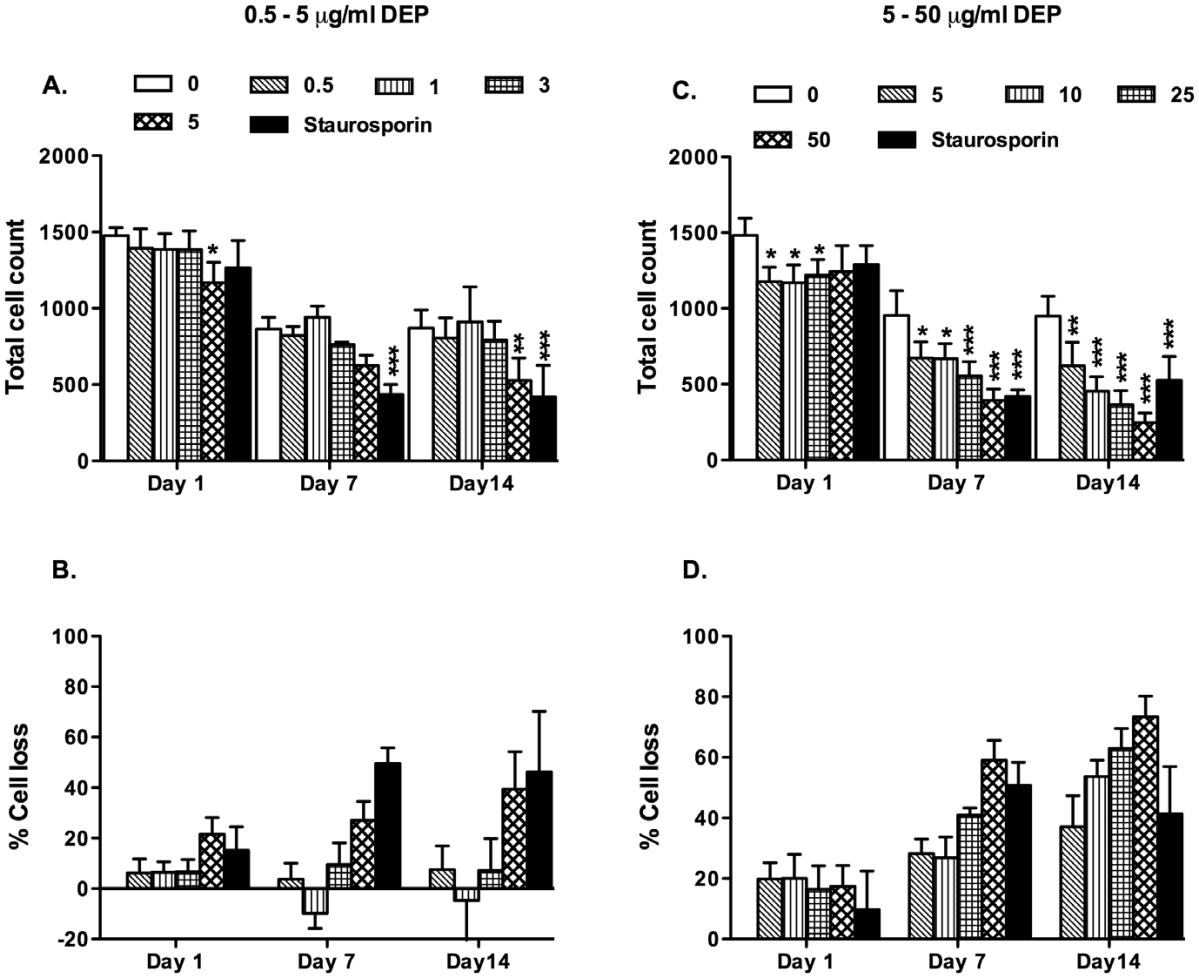
DEP induced cell loss of MDMs from healthy volunteers. MDMs were differentiated from purified monocytes obtained from healthy volunteers in the presence or absence of varied concentrations of DEP as described in the methods. A control well of MDMs were incubated overnight in 1 µM staurosporine (ST). MDMs were visualised by microscopy at the stated time points. A total of 4 randomly selected 10×magnification fields were visualised and cell counts were performed and denoted as total cell count. Data shown are mean±SEM of n = 6 for panels A and B and n = 4 for C and D, performed on different donors. Significant differences in A and B are denoted by *p<0.05, **p<0.01 and ***p<0.001, compared to MDMs differentiated in the absence of DEP, as measured by two way ANOVA and Bonferroni’s post test. To facilitate comparison the percentage cell loss was calculated compared to MDMs differentiated in the absence of DEP (C and D).

We therefore investigated whether MDMs differentiated in the presence or absence of DEP, from normal donors and people with COPD, showed alterations in their phenotype and function that might have implications for the pathology of particulate-induced airways disease.

**Figure 2:**
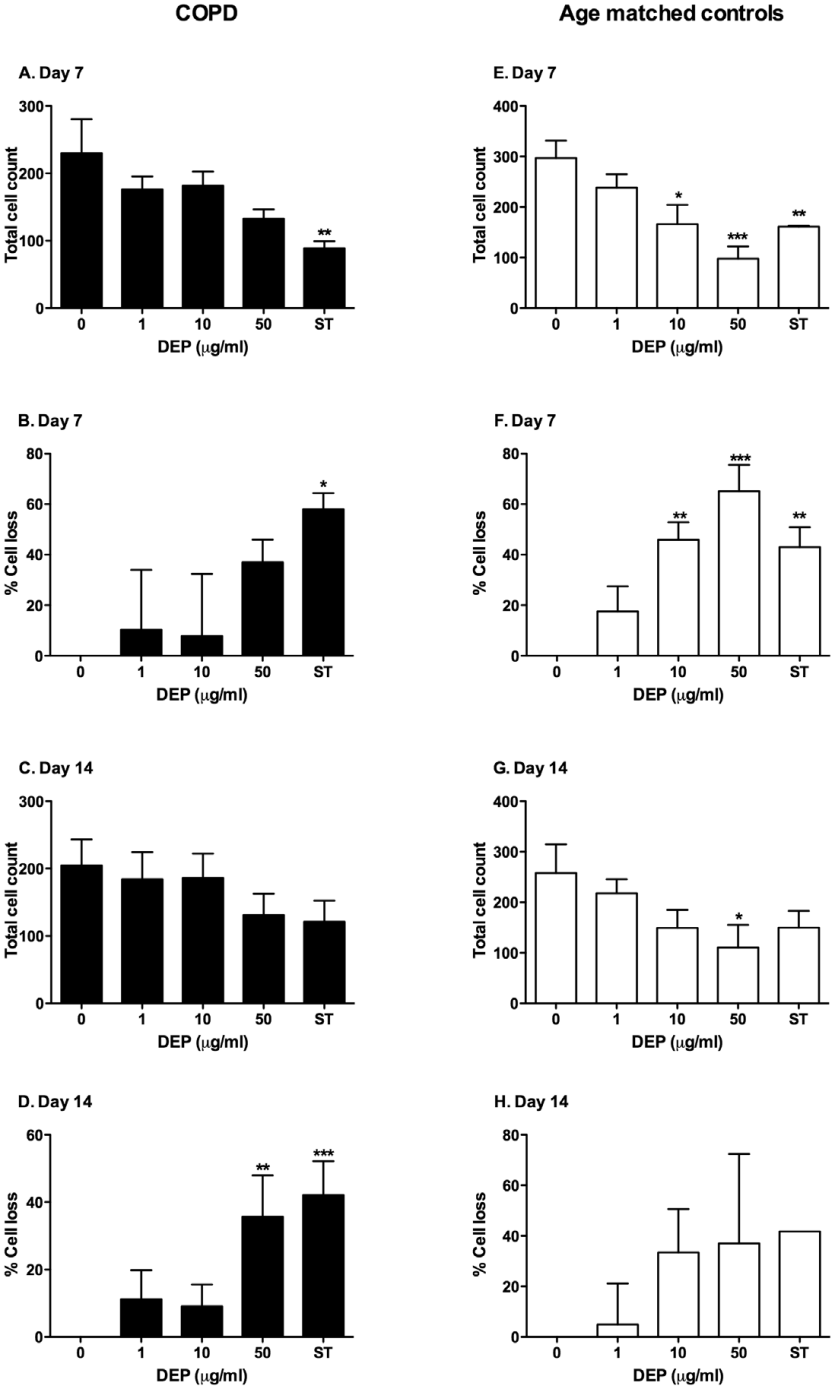
DEP induced cell loss of MDMs from COPD and age matched controls. MDMs were differentiated from purified monocytes obtained from stage II GOLD criteria COPD and age matched controls in the presence or absence of varied concentrations of DEP as described in the methods. A control well of MDMs was incubated overnight in 1 µM staurosporine (ST). MDMs were visualised by microscopy at the stated time points. A total of 4 randomly selected 40 magnification fields were visualised and cell counts were performed and denoted as total cell count. Data shown are mean±SEM of n = 4, performed on different donors with significant differences denoted by *p<0.05, **p<0.01 and ***p<0.001 compared to MDMs differentiated in the absence of DEP, as measured by one way ANOVA and Dunnett’s post test. To facilitate comparison the percentage cell loss was calculated compared to MDMs differentiated in the absence of DEP.

## Materials and Methods

### Materials

Reagents were purchased from Sigma-Aldrich (Poole, UK) or Invitrogen (Paisley, UK), except where specified. National Institute of Standards and Technology Diesel Exhaust Particles were used (NIST DEP SRM 2975).

**Figure 3:**
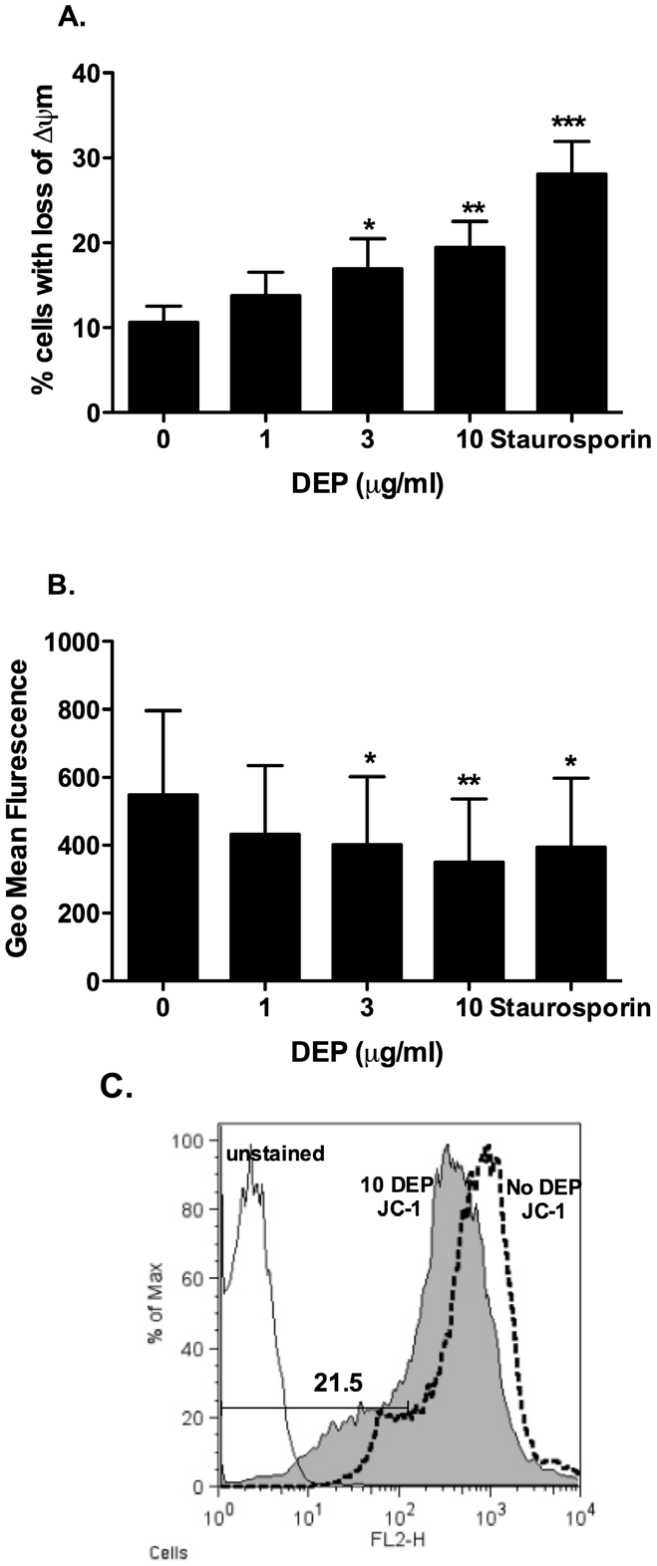
MDMs differentiated in the presence of DEP showed loss of mitochondrial membrane potential (ΔΨm). MDMs were differentiated from purified monocytes in the presence or absence of varied concentrations of DEP as described in the methods. At day 14 MDMs and DEP-MDMs from healthy volunteers were washed three times in PBS. Cells were stained with 10 µM JC-1 in serum free RPMI 1640 for 30 minutes at 37°C. The cells were harvested and resuspended in ice cold PBS for analysis by flow cytometry. Loss of Δψ_m_ was detected using a FACSCalibur flow cytometer and was indicated by a decrease in red fluorescence (FL-2). Viable MDMs were identified based on size and granularity on the forward and side scatter plots and ten thousand events were recorded. Data were presented as percentage of cells showing loss of red staining compared to control cells (A) and geomean fluorescence (B) and data analysis was performed using FlowJo software. Figure C shows a representative histogram flow analysis plot from one donor. Data shown are mean±SEM of n = 4–5, performed on different donors with significant differences denoted by *p<0.05, **p<0.01 and ***p<0.001, compared to MDMs differentiated in the absence of DEP, as measured by one way ANOVA and Dunnett’s post test.

### Monocyte Preparation

All studies involving healthy human volunteers were approved by the Sheffield Research Ethics Committee and authorised and sponsored by Sheffield Teaching Hospitals NHS Foundation Trust (protocol STH15395, Sheffield Research Ethics Committee Ref 05/Q2305/4). Studies involving Stage II GOLD criteria COPD and age matched controls were approved by the Northwest Research Ethics Committee (protocol 10/H1016/25) and sponsored by the Medicines Evaluation Unit Ltd (protocol DS­10­01). All studies required fully informed written consent in full accordance with all the principles of the Declaration of Helsinki.

**Figure 4:**
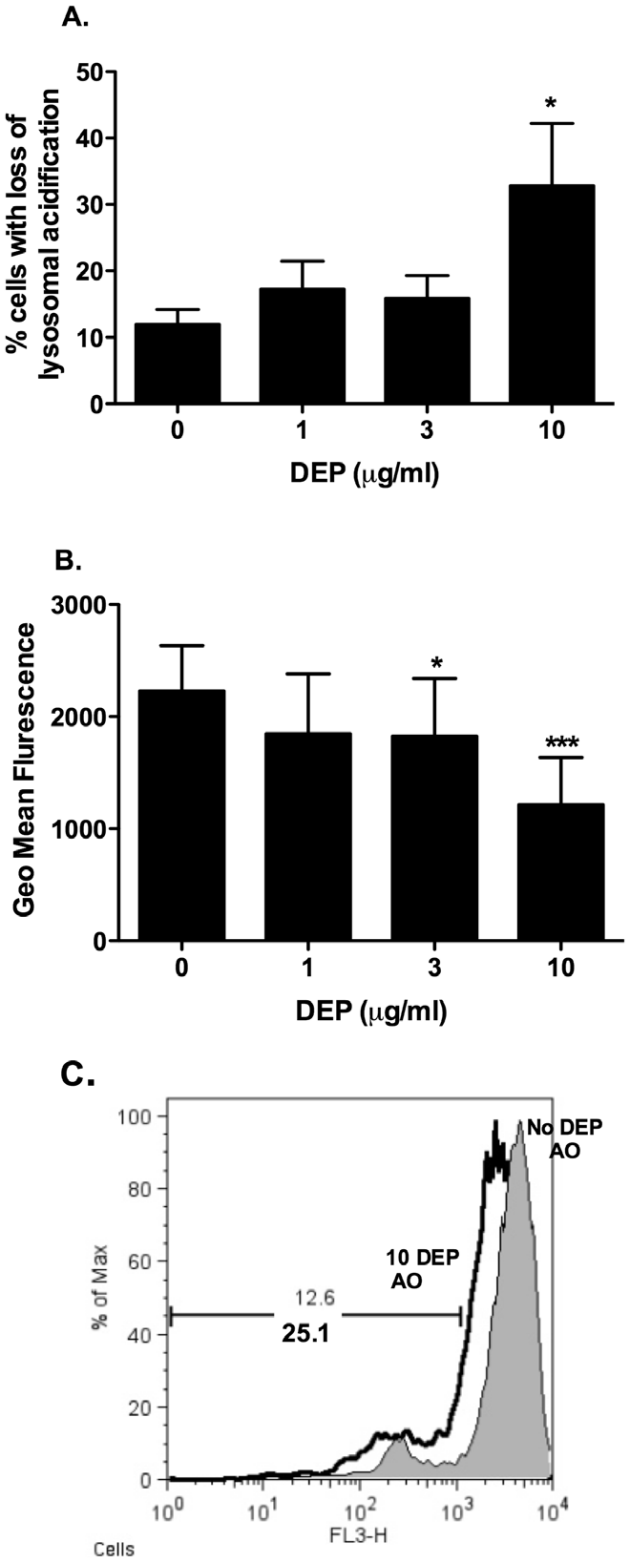
MDMs differentiated in the presence of DEP showed loss of lysosomal acidification. MDMs were differentiated from purified monocytes in the presence or absence of varied concentrations of DEP as described in the methods. At day 14 cells from healthy volunteers were stained with 5 µM acridine orange (AO) for 30 minutes at 37°C. The cells were harvested and resuspended in ice cold PBS for analysis using a FACSCalibur flow cytometer. Loss of lysosomal acidification was measured in the FL-3 channel. Viable MDMs were identified based on size and granularity on the forward and side scatter plots and ten thousand events were recorded. Data were presented as percentage of cells showing loss of green staining compared to control cells (A) and geomean fluorescence (B) and data analysis was performed using FlowJo software. Figure C shows a representative histogram flow analysis plot from one donor. Data shown are mean±SEM of n = 4–5, performed on different donors with significant differences denoted by *p<0.05, **p<0.01 and ***p<0.001, compared to MDMs differentiated in the absence of DEP, as measured by one way ANOVA and Dunnett’s post test.

Initial investigations were performed on healthy volunteers as proof of concept and then selected experiments were further explored in individuals with Stage II GOLD criteria COPD and age matched controls (Table 1). Peripheral blood mononuclear cells (PBMCs) were separated from granulocytes by centrifugation over density gradients using OptiPrep (Axis shield, Oslo, Norway) as previously described [20]. Monocytes were further purified by negative magnetic selection using the Monocyte Isolation Kit II (Miltenyi Biotec, Bergisch Gladbach, Germany) to a purity of approximately 91±0.5% (mean±SEM) CD14^+^ cells.

**Figure 5:**
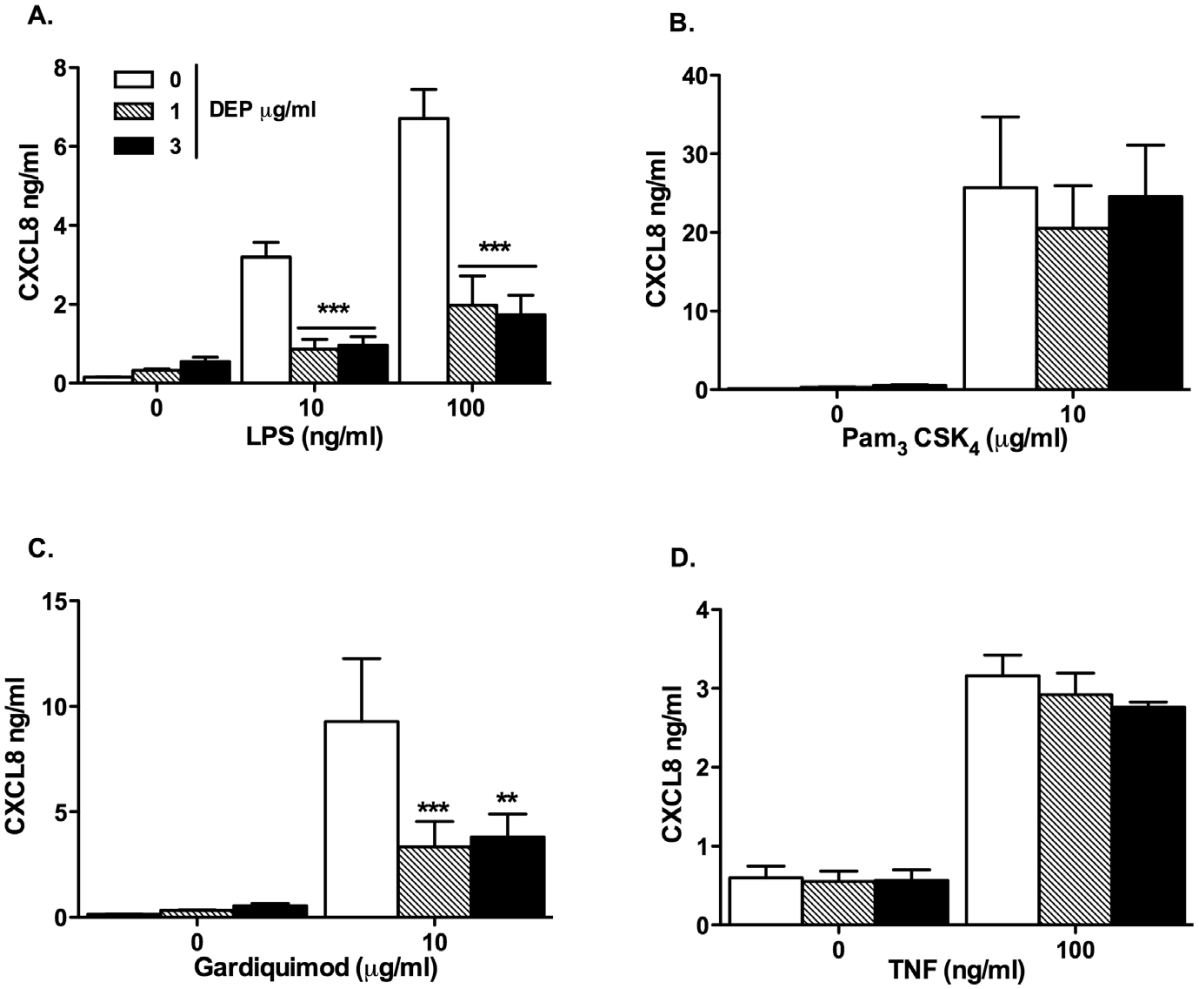
MDMs from healthy volunteers differentiated in the presence of DEP showed reduced proinflammatory responses to LPS. MDMs from healthy volunteers were differentiated from purified monocytes in the presence or absence of varied concentrations of DEP as described in the methods. At day 14 MDMs were washed and stimulated with varied TLR agonists or TNFα as shown. After 24 hours, levels of CXCL8 in the supernatant were determined by ELISA. Data shown are mean±SEM of n = 4, performed on different donors with significant differences denoted by *p<0.05, **p<0.01 and ***p<0.001, compared to MDMs differentiated in the absence of DEP, as measured by two way ANOVA and Bonferroni’s post test.

### Timelapse Microscopy

Purified monocytes from healthy volunteers, at 2.5×10^6^/ml, were stimulated with 50 µg/ml DEP for 1 hour. During this time cells were visualised using an Olympus IX/71 inverted microscope and timelapse microscopy was performed using a Coolsnap HQ/ICX285 monochrome camera capturing images every 30 seconds over a period of 60 minutes.

**Figure 6:**
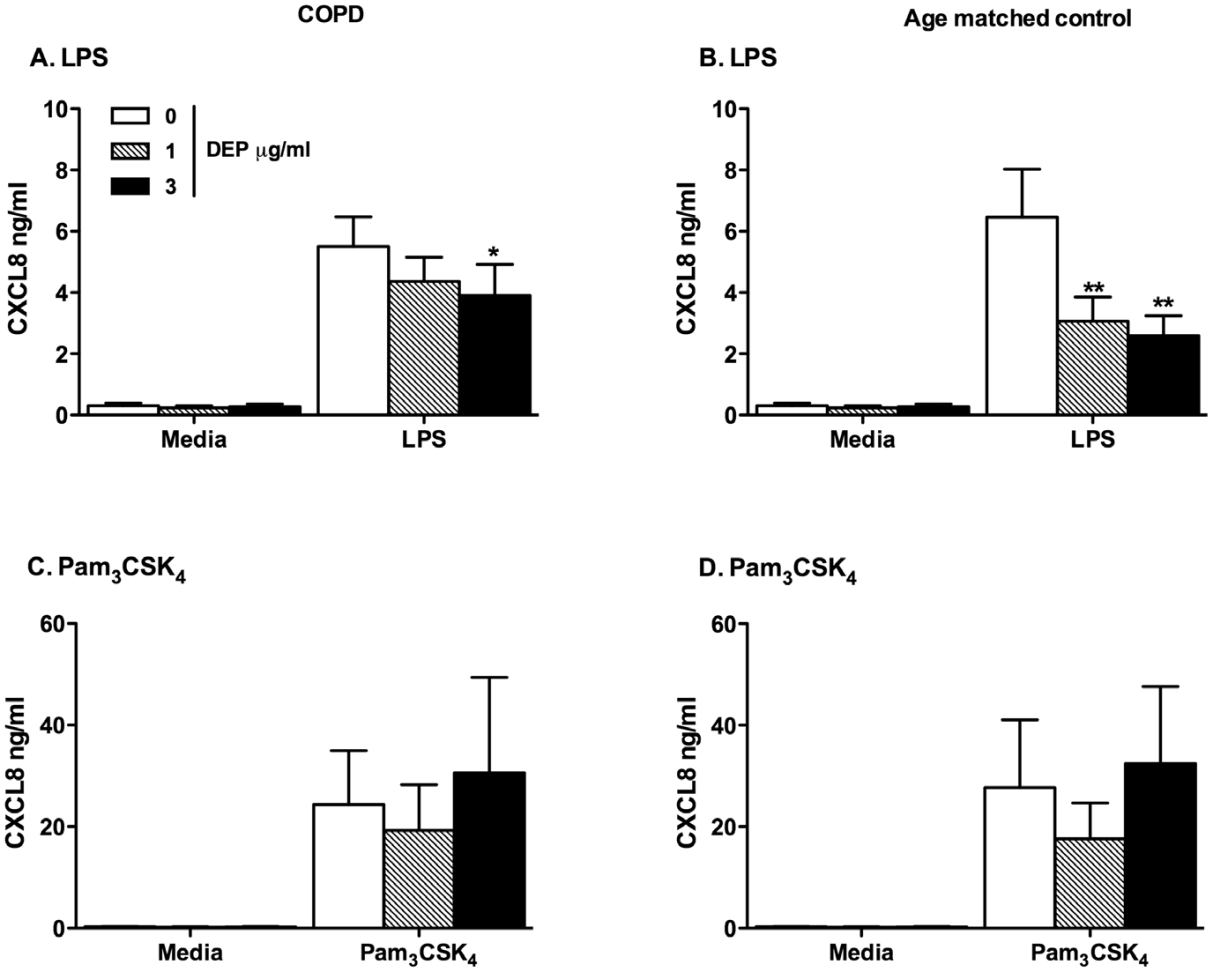
MDMs from COPD and age matched controls differentiated in the presence of DEP showed reduced proinflammatory responses to LPS. MDMs from COPD and age matched controls were differentiated from purified monocytes in the presence or absence of varied concentrations of DEP as described in the methods. At day 14 MDMs were washed and stimulated with 10 ng/ml LPS or 10 µg/ml PAM_3_CSK_4_. After 24 hours, levels of CXCL8 in the supernatant were determined by ELISA. Data shown are mean±SEM of n = 5, performed on different donors with significant differences denoted by *p<0.05, **p<0.01 and ***p<0.001, compared to MDMs differentiated in the absence of DEP, as measured by one way ANOVA and Dunnett’s post test.

### Preparation of Monocyte-Derived Macrophages (MDM)

Monocytes were isolated and purified as described above. Cells were serum starved for 1 hour in 12 well tissue culture plates at 250,000/well in RPMI 1640 with 1% penicillin G (100 units/ml) and streptomycin (100 µg/ml). The media was then removed and cells washed with phosphate buffered saline (PBS) to remove non-adherent cells. The media was replaced with RPMI 1640 with 10% newborn calf serum and penicillin G (100 units/ml) and streptomycin (100 µg/ml) (MDM media) with 50 ng/ml macrophage colony stimulating factor (M-CSF) (PeproTech EC Ltd, London, UK). Cells were cultured for three to four days and the media then replaced with MDM media without M-CSF. Cells were cultured for a total of 14 days with cell washes and media changes every three to four days, allowing differentiation of monocytes into MDM. For MDM that were differentiated in the presence of DEP (DEP-MDM), varied concentrations of DEP were added to the cells at the onset of culturing and at each change of media.

**Figure 7:**
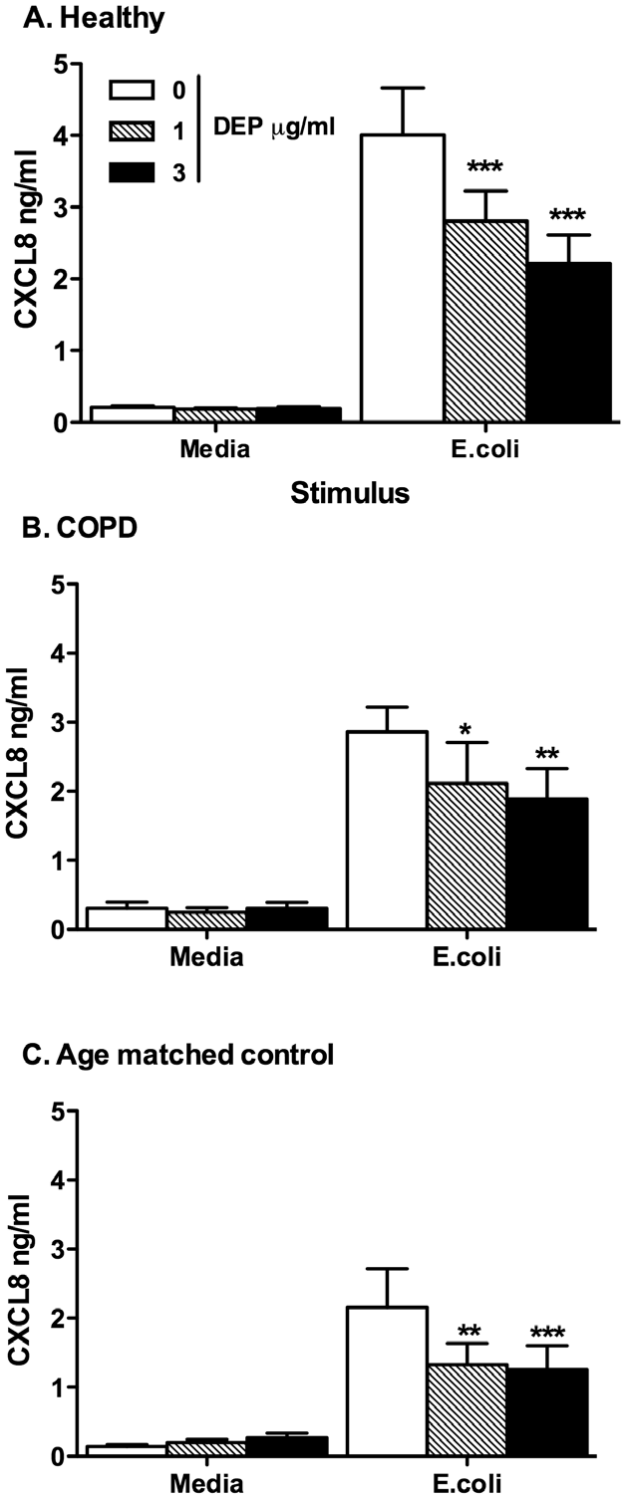
MDMs from healthy volunteers differentiated in the presence of DEP showed reduced proinflammatory responses to *E. coli*. MDMs from healthy volunteers, COPD and age matched controls were differentiated from purified monocytes in the presence or absence of varied concentrations of DEP as described in the methods. At day 14 MDMs were washed and stimulated with heat killed *E. coli* as shown. After 24 hours, levels of CXCL8 in the supernatant were determined by ELISA. Data shown are mean±SEM of n = 5, performed on different donors with significant differences denoted by *p<0.05, **p<0.01 and ***p<0.001, compared to MDMs differentiated in the absence of DEP, as measured by one way ANOVA and Dunnett’s post test.

At the indicated time points MDMs and DEP-MDMs were harvested by washing the cells in PBS and harvested by gently scraping using a cell scraper.

**Figure 8:**
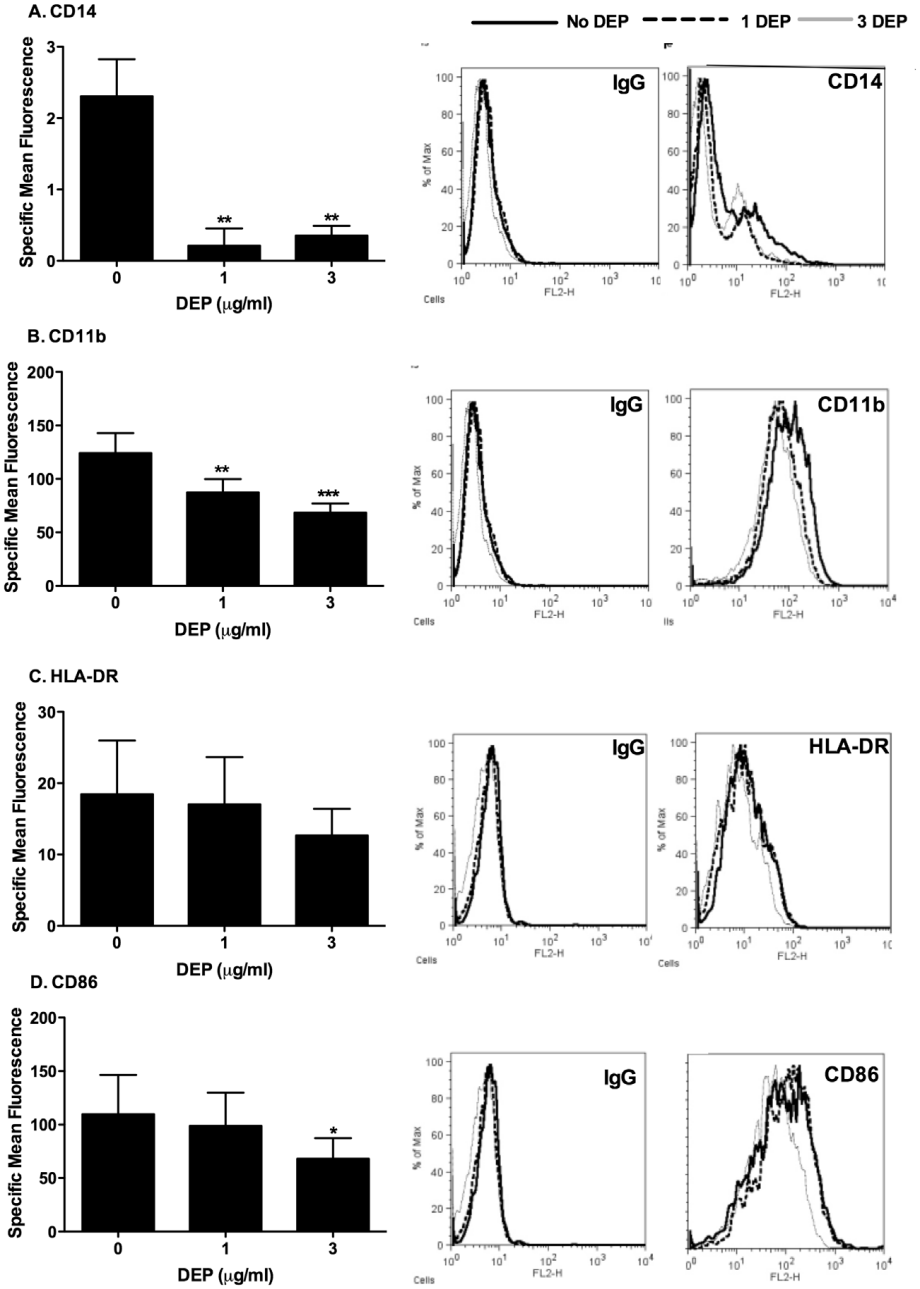
MDMs differentiated in the presence of DEP showed reduced CD14, CD11b and CD86 surface marker expression. MDMs from healthy volunteers were differentiated from purified monocytes in the presence or absence of varied concentrations of DEP as described in the methods. At day 14 MDMs were harvested and non specific binding was blocked by incubating at room temperature for 10 minutes with 1∶50 IgG from murine serum reagent grade in FACS buffer. Cells were then incubated with 1∶10 PE conjugated mouse anti-human CD14, CD11b, HLA-DR, TLR2, TLR4, CD80 or CD86 or the respective isotype controls. PE fluorescence was measured on the FL-2 channel of a FACSCalibur flow cytometer. Data are presented as a mean±SEM of n = 4 performed on different donors and representative histogram flow analysis plots from one donor. Significant differences between MDMs and DEP-MDMs cell surface expression are denoted by *p<0.05 and **p<0.01 as measured by one way ANOVA and Dunnett’s post test.

### Assessment of Cell Loss

At day 1, 7, and 14, MDMs and DEP-MDMs were washed three times with PBS and the media replaced. 10 µM Hoechst was added to the wells and incubated in the dark for three minutes. Cells were visualised using a fluorescence and phase contrast Leica DM14000B inverted microscope. For healthy volunteers a total of 4 randomly selected 10× magnification fields were visualised and cell counts were performed in each field. This resulted in total cell counts ranging from 500–1500. For studies of stage II/III GOLD COPD and age matched controls, and a total of 4 randomly selected 40× magnification fields were visualised. This produced a total cell count within the fields and allowed comparison of cells counts between MDMs and DEP-MDMs.

### Measurement of Loss of Inner Mitochondrial Transmembrane Potential (Δψ_m_)

At day 3 and 14, MDMs and DEP-MDMs from healthy volunteers were washed three times in PBS. Cells were stained with 10 µM 5,5′,6,6′-tetrachloro-1,1′,3,3′-tetraethylbenzimidazolycarbocyanine iodide (JC-1, Molecular Probes, Eugene, OR, USA) in serum free RPMI 1640 for 30 minutes at 37°C. The cells were harvested as described above and resuspended in ice cold PBS. Loss of Δψ_m_ was detected using a FACSCalibur flow cytometer and was indicated by a decrease in red fluorescence (FL-2). Data were quantified as percentage of cells showing loss of red staining compared to control cells [21], analysed by FlowJo software (Tree Star Inc., Ashland, OR, USA).

### Measurement of Loss of Lysosomal Acidification

At day 3 and 14, MDMs and DEP-MDMs from healthy volunteers were washed three times in PBS. Cells were stained with 5 µM acridine orange (AO) in serum free RPMI 1640 for 30 minutes at 37°C. The cells were harvested as described above. Loss of fluorescence was measured by flow cytometry and data were quantified as percentage of cells showing loss of green staining compared to control cells as described [21].

### Treatment of MDMs with Varied TLR Agonists

MDMs and DEP-MDMs from healthy volunteers, individuals with Stage II GOLD criteria COPD, and age matched controls were stimulated at Day 14. At the time of stimulation cells were washed three times in PBS, media was replaced and final volumes in each well of a 12-well plate always totalled 1,000 µl during experiments. Cells were stimulated with TLR agonists: 10 or 100 ng/ml LPS (*E. coli* serotype R515 from Alexis, Nottingham, UK) (TLR4), 10 µg/ml Pam_3_CSK_4_ (TLR2) or 10 µg/ml gardiquimod (Invivogen, Toulouse, France) (TLR7/8). Controls were stimulated with 10 ng/ml TNFα. Stimulated cells were incubated for 24 hours at 37°C and humidified 5% CO_2_. Cell-free supernatants were prepared and stored at –80°C until cytokine generation was determined.

### Treatment of MDMs with *E. coli*

MDMs and DEP-MDMs from healthy volunteers, individuals with Stage II GOLD criteria COPD, and age matched controls were stimulated at Day 14 as described above. Cells were stimulated with heat killed *E. coli* at a ratio of 100 heat killed bacteria per cell for 24 hours at 37°C. Cell-free supernatants were prepared and stored at –80°C until cytokine generation was determined.

### Enzyme Linked Immunosorbent Assay (ELISA)

Cell-free supernatants were prepared and stored at –80°C until cytokine generation was determined by ELISA using matched pairs of antibodies (R&D Systems, Abingdon, UK) at optimized concentrations as previously described [22]. Absorbance was measured at 450 nm using an MRX plate reader (Thermo Labsystems, Vantaa, Finland) and Biolinx software version 2.20 (Biolinx, Frankfurt am Main, Germany). Samples were typically diluted so that the optical density fell within the optimal portion of a log-lin standard curve. Limits of detection (LD) for the ELISAs were 31.25 pg/ml. Samples whose values were below the LD were assigned the LD value for analysis.

### Phagocytosis of Latex Beads

Red fluorescent carboxylate- modified latex beads with a mean diameter of 2 µm (L3030, Sigma) were opsonised in 10% human AB serum (serum isolated from healthy volunteers) with RPMI 1640 for 60 minutes at 37°C and then incubated at an MOI of 10 with washed MDM and DEP-MDMs from healthy volunteers for 4 hours to allow phagocytosis. To account for extracellular binding, phagocytosis was inhibited by adding 5 µM cytochalasin D to control samples for 30 minutes prior to adding beads. Cells were washed three times in PBS and harvested as described previously. Phagocytosis was determined using flow cytometry by detecting an increase in fluorescence associated with internalised beads. The median fluorescence intensity of samples containing cytochalasin D was subtracted from samples exposed to beads in the absence of cytochalasin D.

### Cell Surface Marker Expression by Flow Cytometry

MDMs and DEP-MDMs from healthy volunteers were harvested at day 3 and 14 as described. Cells were suspended in MDM media, centrifuged at 1000 *g* for 3 minutes and resuspended in buffer (PBS, 0.25% bovine serum albumin, 10 mM HEPES). The cells were incubated at room temperature for 10 minutes with 50 µg/ml murine IgG and then incubated with 1∶10 PE-conjugated anti-human TLR2 (Clone TL2.1), TLR4 (Clone HTA125), CD11b (Clone ICRF44), CD14 (Clone 61D3), HLA-DR (Clone LN3), CD80 (B7-1) or CD86 (B7-2) antibody or their relevant 1∶10 PE-conjugated mouse IgG2a (TLR2 and TLR4), IgG2b (HLA-DR and CD86) and IgG1 (CD14, CD11b and CD80) isotype controls (eBioscience, San Diego, USA). Fluorescence was measured in the FL-2 channel of a FACSCalibur flow cytometer. Ten thousand events were recorded and data analysis was performed using FlowJo software (Tree Star Inc., Ashland, OR, USA) and quantified as specific mean fluorescence.

### Statistics

Data were analysed using GraphPad Prism v5 (GraphPad Inc, San Diego, CA, USA). Data were compared using one-way or two-way analysis of variance (ANOVA) with the appropriate post tests with a statistically significant values of p<0.05.

## Results

We have previously demonstrated the important role that monocytes can play in the cooperative signalling with epithelial cells in response to DEP and pathogenic stimuli [23]. In active inflammation monocytes are recruited to the airways and can replace resident alveolar macrophages [14]. We therefore investigated whether chronic low dose DEP exposure can alter monocyte phenotype or function. Here we have shown that monocytes interact with and phagocytose DEP and that chronic DEP exposure has profound effects on monocyte functional status.

### DEP Induces Significant Cell Loss of MDMs Over 14 Days Differentiation

We first studied the interaction of monocytes with DEP by examining the extent to which they phagocytosed these particles. Although difficult to quantify, in previous unpublished work we have found by light microscopy that after a period of 24 hour exposure monocytes contain phagocytosed DEP within vacuoles. We demonstrate here that over a 1 hour exposure, monocytes avidly interact with DEP in their vicinity (Figure S1 and S2). Even over this short time period, a majority of visible DEP are either phagocytosed or bound to monocytes. We therefore sought to determine how persistent interaction of monocytes with DEP would alter their survival or function.

In healthy volunteers, compared to untreated cells, those differentiated in the presence of DEP concentrations greater than 5 µg/ml demonstrated a marked, significant reduction in absolute cell number over a period of 14 days (Figure 1). Similarly in individuals with stage II/III GOLD criteria COPD and age matched controls, higher concentrations of DEP caused a reduction of absolute cell numbers reaching statistical significance in age matched controls at 7 days (Figure 2). Monocytes from individuals with COPD were more resistant to the effects of DEP compared to age matched controls. 10 µg/ml of DEP had no significant impact on cell numbers at day 7 in COPD individuals, but in age-matched controls, the same dose of DEP caused significant cell loss at this time point. Cell loss in cells from people with COPD did not achieve statistically significant levels.

### MDMs Differentiated in the Presence of DEP Cause Loss of Mitochondrial Membrane Potential (ΔΨm)

The significant cell loss observed over time with higher concentrations of DEP led us to investigate the potential mechanisms in healthy volunteers. We initially looked at inner mitochondrial membrane permeabilisation, a feature associated with mitochondrial dysfunction and cell death pathways including apoptosis. Here we demonstrate that DEP concentrations of 3 and 10 µg/ml caused a loss of inner mitochondrial membrane potential at day 14 (Figure 3). Similar changes were seen at earlier time points (3 days), but at these earlier points when macrophage differentiation was incomplete, cell fragility precluded reliable recovery of cells for statistical analysis.

### MDMs Differentiated in the Presence of DEP Cause Loss of Lysosomal Acidification

We sought to ascertain whether monocyte uptake of DEP had an effect on lysosomal function. Loss of lysosomal acidification was detected using AO as described in the methods. Here we show in healthy volunteers that higher concentrations of DEP at 10 µg/ml cause loss of lysosomal acidification at day 14. Lower concentrations of DEP (1 and 3 µg/ml) had no effect (Figure 4). Similar changes were seen at earlier time points (3 days), but at these earlier points when macrophage differentiation was incomplete, cell fragility precluded reliable recovery of cells for statistical analysis.

### MDMs Differentiated in the Presence of DEP did not Demonstrate Cytoplasmic Release of Cytochrome c or Caspase Activation

Loss of inner mitochondrial membrane potential can result in the release of cytochrome c into the cytoplasm and subsequent activation of various caspases. Because we demonstrated a loss of ΔΨm with DEP differentiated cells we hypothesised that there would be evidence of cytoplasmic release of cytochrome c and subsequent caspase activation. However we found by western blot analysis that this was not detected in the presence of DEP (data not shown).

### MDMs Differentiated in the Presence of DEP Showed Reduced Responses to TLR Agonists

DEP exposure caused monocyte loss, and we also therefore considered that DEP would alter monocyte function. We elected to study functional responses at concentrations of DEP not causing significant cell loss to facilitate accurate comparisons between groups. In healthy volunteers (Figure 5), or individuals with COPD and their age matched controls (Figure 6), MDMs differentiated in the presence of low concentrations of DEP produced markedly less CXCL8 in response to LPS (TLR4) and gardiquimod (TLR7), but similar levels in response to Pam_3_CSK_4_ (TLR2) and TNFα.

### MDMs Differentiated in the Presence of DEP Showed Reduced Responses to *E. coli*

The altered functional response of reduced cytokine release, primarily to LPS, was further explored by stimulating cells from healthy individuals, those with COPD and age matched controls with heat killed bacteria *E. coli*. Similarly to the findings with LPS, MDMs from all 3 groups differentiated in the presence of DEP produced less CXCL8 in response to heat killed *E. coli* (Figure 7). We observed in healthy volunteers that DEP exposure did not alter phagocytosis of latex beads (data not shown).

### MDMs Differentiated in the Presence of DEP Showed Altered TLR4 Coreceptor Expression

Chronic DEP exposure may affect macrophage differentiation and it was proposed that this reduction in CXCL8 response to LPS and heat killed bacteria may be due to altered states of differentiation. We examined the expression of 7 surface markers by flow cytometry in healthy volunteers. At day 14 there was no detectable surface CD80 expression and very low surface TLR2 and TLR4 expression that was unaltered in the presence of DEP (data not shown). In contrast, CD14 was expressed at low levels that were further reduced by DEP exposure, and CD11b and CD86 were expressed at high levels that were modestly but significantly reduced by DEP (Figure 8).

## Discussion

In this study we have identified additional effects that DEP exposure may have on the function of monocytic phagocytes, with potential consequences for pulmonary innate immunity. In particular, we have shown that chronic treatment of cells with DEP results in cellular toxicity with marked loss of cells in prolonged culture. In addition chronic low dose diesel particulate treatment that is at levels thought to be representative of environmental pollution exposure impaired the proinflammatory responses of monocyte-derived macrophages to pathogenic stimuli.

Studies have demonstrated that alveolar macrophages and MDMs from COPD individuals show reduced phagocytosis of apoptotic epithelial cells [17] and reduced phagocytosis of pathogens [18] respectively, compared to non smokers. Epithelial cells from individuals with COPD show higher susceptibility to the effects of cigarette smoke with the release of the proinflammatory mediators IL-1β and ICAM-1, and reduction of antioxidant levels [24]. The nasal epithelium of individuals with severe COPD also have reduced TLR4 expression [19] and expression of IL-1 receptor antagonist (IL-1ra) [25], which could have major implications for airway responses to inhaled pathogens and the subsequent development of chronic inflammation [19]. In light of these findings our aims were two fold. Firstly, to explore the chronic effects of DEP exposure on monocyte phenotype and function in normal healthy individuals. Secondly, to address the hypothesis that MDMs from COPD individuals would have altered responses to DEP.

As we show here, monocytes avidly interact with and phagocytose DEP, and we have previously shown that monocytes are important mediators of cooperative inflammatory responses with epithelial cells in response to DEP [23]. In our studies in healthy volunteers chronic exposure to higher concentrations of DEP (>10 µg/ml) caused loss of lysosomal acidification and loss of mitochondrial membrane polarisation, and was associated with a gradual loss of cells over time both in healthy and COPD individuals. We observed that chronic exposure to concentrations of DEP that are generally perceived to be representative of low level exposure can impair responses to pathogenic stimuli. Finally, we demonstrate a reduction in cell surface expression of receptors and molecules that are implicated in these responses. These data demonstrate that the actions of chronic DEP exposure may impair the maintenance of a stable macrophage environment in the lungs of healthy and COPD individuals, with potentially important consequences in inflammatory lung diseases and control of pathogens.

Extrapolation of *in vitro* concentrations to *in vivo* exposure is complicated by a number of physiological factors such as variations in tidal breathing, body size, lung surface area and clearance mechanisms. Using a complex *in vivo* dosimetric study Li *et al* has equated a concentration range of 1–100 µg/ml to pollution levels experienced over 24 hours in a California state [26]. The majority of *in vitro* and *in vivo* studies exploring the inflammatory effects of DEP utilise concentrations above 50 µg/ml, with a dose dependent effect on varied cell types [4], [6], [10], [15], [27], [28], [29], [30]. We have observed here that lower concentrations over longer time periods can have important effects on monocyte function, potentially extending the dose range at which DEP exposure may have important physiological effects, and suggesting that chronicity of exposure may be important in understanding pathological consequences.

The direct toxicity of DEP over short time courses in a variety of cell types is well established [6], [12], [27], [28], [29], [30]. Here we demonstrate that chronic exposure over 14 days to concentrations of DEP>5 µg/ml induced a marked loss of monocytes during their differentiation to macrophages, from both healthy and COPD individuals. Monocytes from people with COPD were more resistant to DEP-induced loss, since cell loss did not achieve statistical significance at the earlier time point and only achieved significance with higher doses of DEP at later points. The mechanisms underpinning this partial resistance to cell loss remain to be determined, but may reflect some aspect of chronic inflammatory conditioning of these cells. A limitation of our study is that we only had one control group for people with COPD, these being healthy age-matched non smokers. Thus we cannot be clear as to whether the differences we have observed are a result of smoking, COPD, or both processes. We observed that cell loss in healthy individuals was associated with a loss of mitochondrial membrane potential, a feature consistent with induction of apoptosis. In human macrophages and macrophage cell lines DEP has previously been shown to induce a concentration and time dependent increase in apoptosis with mitochondrial dysfunction, though these studies used higher concentrations of DEP over a shorter 10 hour time period [11]. We attempted to define mechanisms of cell loss in more detail, measuring cytochrome c release and caspase activation by western blotting. However, at any time point of the assay the observed cell loss equated to a 2.9–5.7% cells lost per day or 0.12–0.24% per hour. We were unable to detect cytochrome c release and caspase activation in cells chronically exposed to all concentrations of DEP, probably consequent upon the small number of cells at any point executing the final components of the cell death pathways. It may also be that activation of these cell death pathways would also be rapidly associated with detachment from the plate, and such non-adherent cells would not be captured in our assays. We considered including additional assays such as TUNEL to measure cell loss, but again the very low hourly rate of cell death would make detecting changes in cell death rates with this assay very challenging.

The marked loss of cells during differentiation with higher concentrations of DEP led us to investigate the functional consequences of DEP exposure. We studied cellular responses to chronic exposure to low concentrations of DEP for two reasons, firstly higher concentrations of DEP caused cell loss that would confound our results and secondly we hypothesised that changes in cell phenotype were likely to occur at concentrations of DEP less than those inducing cell death. We found that MDMs from healthy and COPD individuals differentiated in the presence of low concentrations of DEP had reduced CXCL8 production in response to the TLR4 agonist LPS and the synthetic TLR7/8 agonist gardiquimod, but not the TLR2 agonist PAM_3_CSK_4_ or the cytokine TNFα. The phagocytosis of latex beads was preserved and the same in MDMs and DEP-MDMs at day 14 suggesting that the reduction in CXCL8 production in DEP-MDMs could not be entirely explained by overload of the MDMs by DEP.

We also showed that monocytes avidly interact with and phagocytose DEP, and this may result in lysosomal dysfunction. Reduced responses to gardiquimod suggest that endosomal signalling may be disrupted as a result of DEP uptake. Interestingly, TLR4 signalling has components that are activated at the cell membrane (via MyD88) and the endosome (via TRAM) [31]. The preservation of TLR2 signalling and responses to TNFα may suggest that responses at the cell membrane are relatively preserved, and a feasible explanation for the reduction of responses to LPS may be that a component of the TLR4 signal from the endosome is impaired similarly to the loss of responses to gardiquimod. Alternatively, disruption of endosomal function may alter TLR4 localisation and trafficking, which are also important considerations in the generation of an effective TLR4 response [32]. A final mechanism potentially reducing TLR4 signalling is loss of essential coreceptors such as CD14 [33] or CD11b [34]. Our data show a loss of cell surface CD14 and CD11b expression in DEP-exposed monocytes. The relative contribution of each of these pathways – altered signalling from the endosomal compartment, altered TLR4 trafficking, or loss of coreceptors – in the downregulation of TLR4 signalling is our current target for exploration. Future work will also focus on the extension of work to a more detailed characterisation on macrophage M1/M2 phenotypes, and whether DEP exposure changes the proportion of cells showing wound healing vs proinflammatory phenotypes.

Consistent with our findings with LPS, we observe a reduction of CXCL8 production in MDMs differentiated in the presence of DEP in response to heat killed *E. coli*, a Gram negative bacteria, that activates TLR4 signalling. In combination with evidence that DEP alters macrophage responses to subsequent bacterial and viral pathogen invasion [35], [36], our data provide additional potential explanations for the observed positive association between living next to a heavily lorry-trafficked road and the development of lower respiratory tract infection (LRTI), bronchiolitis and pneumonia [9].

We observed no differences between healthy age matched controls and individuals with COPD in CXCL8 production in response to TLR agonists or *E. coli.* These results contrast with those of Culpitt et al [37] who found higher CXCL8 production in AM from COPD individuals in response to cigarette smoke extract and LPS, and Taylor et al [18] who showed that MDMs from people with COPD exhibited reduced phagocytosis of pathogens. This may be related to phenotypic differences in our group of COPD individuals who had less severe COPD as measured by FEV1 and fewer pack years of smoking history. It is also true that not all monocytes adhere to plates and differentiate into macrophages, thus it is always possible that subsets of monocytes with different phenotypes have been selectively lost or captured, though these methodological issues remain challenging to measure and control for. Multiple interacting factors such as patient characteristics, underling genetic variations and environmental influences, and minor differences in experimental methodology may therefore account for these variations.

In conclusion our data demonstrate that chronic exposure to low concentrations of DEP exerts multiple detrimental effects on infiltrating monocytes. DEP activates cell networks of monocytes and epithelial cells encouraging the development of inflammation and persistent monocyte recruitment to the airways [23]. Coupled with this, significant death of recruited monocytes and release of toxic mediators could exacerbate and contribute to an already inflamed tissue environment. In addition, disruption of endosomal signalling and alterations of surface marker expression could adversely affect the subsequent responses to pathogenic stimuli. Thus chronic exposure to air pollution in the form of diesel exhaust particles may exacerbate and perpetuate the development chronic inflammation.

## Supporting Information

Figures S1 **Timelapse video: Monocytes rapidly phagocytose DEP.** Purified monocytes were obtained and cultured at 2.5×10^6^/ml as described in the methods. Cells were incubated with 50 µg/ml DEP and timelapse microscopy performed every 30 seconds for 60 minutes as described in the methods. Data shown are representative images of n = 2, performed on different donors.
(MPG)

Figures S2 **Still images: Monocytes rapidly phagocytose DEP.** Methods are described as in Figure S1. Figure S2 are still images of monocytes and DEP at time t = 0 minutes (A) and t = 60 minutes (B) of timelapse microscopy. Data shown are representative images of n = 2, performed on different donors.
(TIFF)
